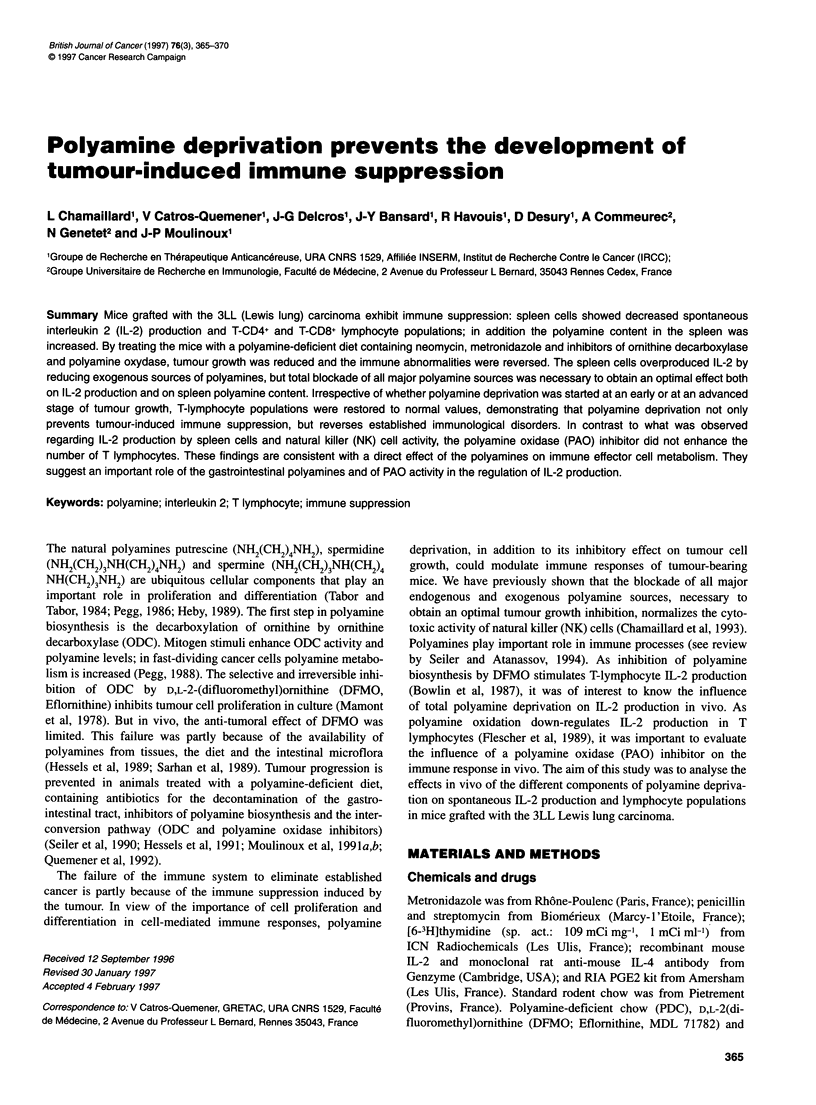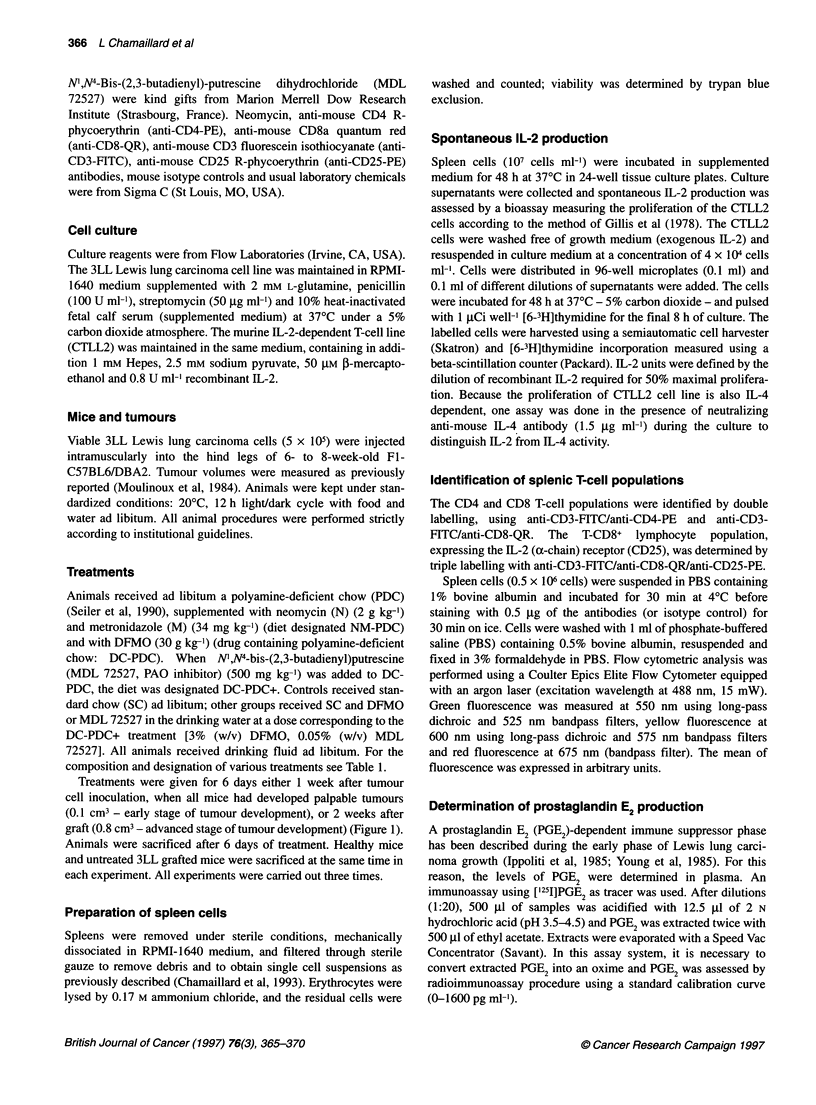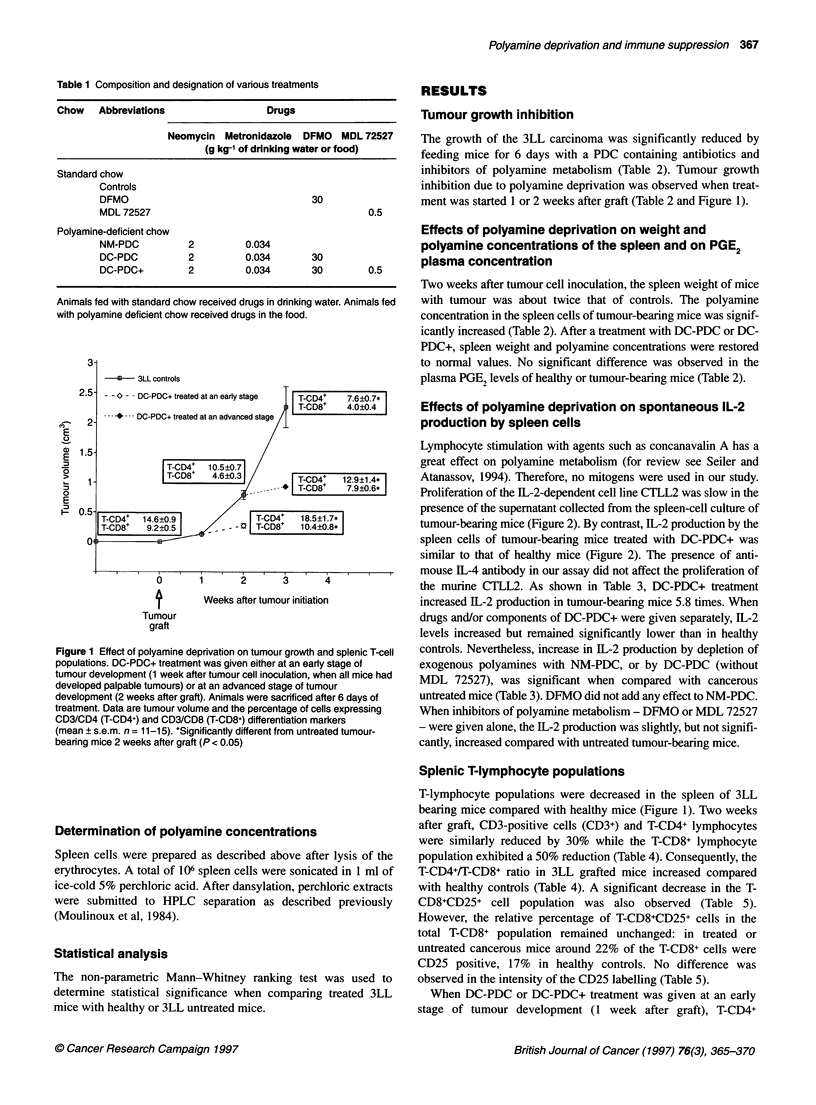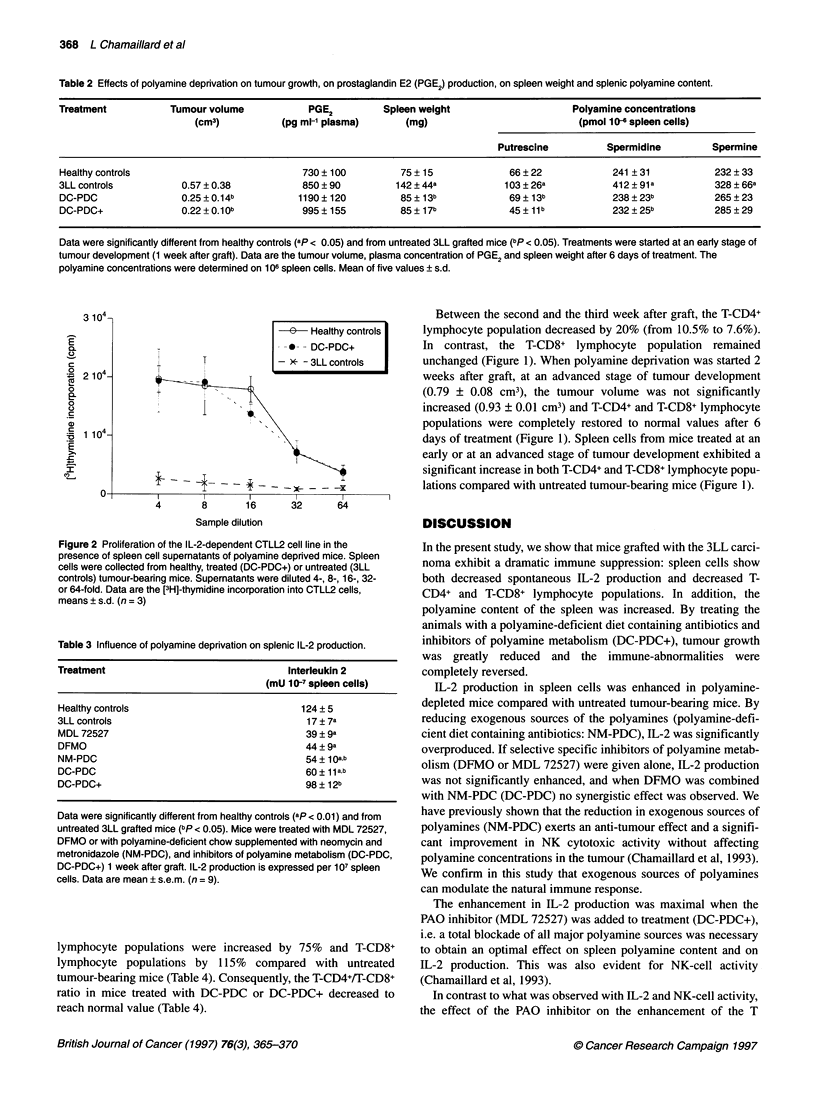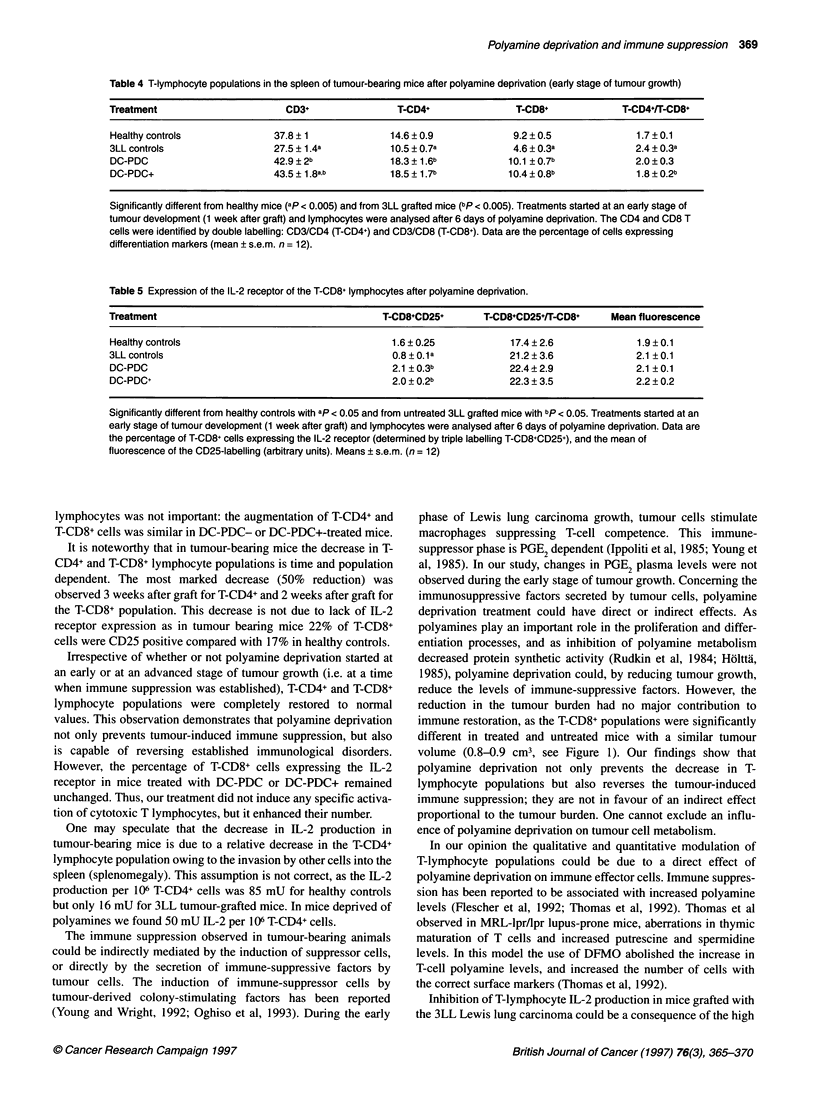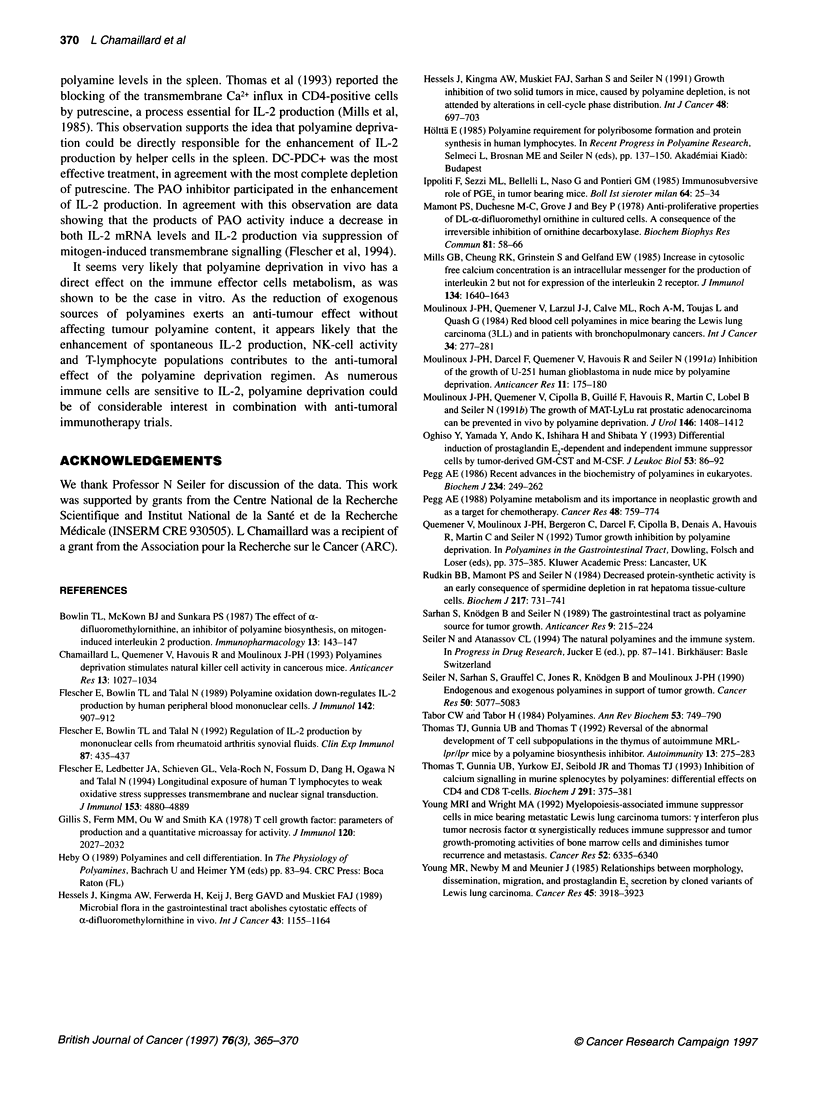# Polyamine deprivation prevents the development of tumour-induced immune suppression.

**DOI:** 10.1038/bjc.1997.391

**Published:** 1997

**Authors:** L. Chamaillard, V. Catros-Quemener, J. G. Delcros, J. Y. Bansard, R. Havouis, D. Desury, A. Commeurec, N. Genetet, J. P. Moulinoux

**Affiliations:** Groupe de Recherche en ThÃ©rapeutique AnticancÃ©reuse, URA CNRS 1529, AffiliÃ©e INSERM, Institut de Recherche Contre le Cancer (IRCC), Rennes, France.

## Abstract

Mice grafted with the 3LL (Lewis lung) carcinoma exhibit immune suppression: spleen cells showed decreased spontaneous interleukin 2 (IL-2) production and T-CD4+ and T-CD8+ lymphocyte populations; in addition the polyamine content in the spleen was increased. By treating the mice with a polyamine-deficient diet containing neomycin, metronidazole and inhibitors of ornithine decarboxylase and polyamine oxydase, tumour growth was reduced and the immune abnormalities were reversed. The spleen cells overproduced IL-2 by reducing exogenous sources of polyamines, but total blockade of all major polyamine sources was necessary to obtain an optimal effect both on IL-2 production and on spleen polyamine content. Irrespective of whether polyamine deprivation was started at an early or at an advanced stage of tumour growth, T-lymphocyte populations were restored to normal values, demonstrating that polyamine deprivation not only prevents tumour-induced immune suppression, but reverses established immunological disorders. In contrast to what was observed regarding IL-2 production by spleen cells and natural killer (NK) cell activity, the polyamine oxidase (PAO) inhibitor did not enhance the number of T lymphocytes. These findings are consistent with a direct effect of the polyamines on immune effector cell metabolism. They suggest an important role of the gastrointestinal polyamines and of PAO activity in the regulation of IL-2 production.


British Journal of Cancer (1997) 76(3), 365–370
© 1997 Cancer Research Campaign

### Polyamine deprivation prevents the development of tumour-induced immune suppression

**L Chamaillard[1], V Catros-Quemener[1], J-G Delcros[1], J-Y Bansard[1], R Havouis[1], D Desury[1], A Commeurec[2], N Genetet[2] and J-P Moulinoux[1]**

[1]Groupe de Recherche en Thérapeutique Anticancéreuse, URA CNRS 1529, Affiliée INSERM, Institut de Recherche Contre le Cancer (IRCC);
[2]Groupe Universitaire de Recherche en Immunologie, Faculté de Médecine, 2 Avenue du Professeur L Bernard, 35043 Rennes Cedex, France

**Summary** Mice grafted with the 3LL (Lewis lung) carcinoma exhibit immune suppression: spleen cells showed decreased spontaneous interleukin 2 (IL-2) production and T-CD4$^+$ and T-CD8$^+$ lymphocyte populations; in addition the polyamine content in the spleen was increased. By treating the mice with a polyamine-deficient diet containing neomycin, metronidazole and inhibitors of ornithine decarboxylase and polyamine oxydase, tumour growth was reduced and the immune abnormalities were reversed. The spleen cells overproduced IL-2 by reducing exogenous sources of polyamines, but total blockade of all major polyamine sources was necessary to obtain an optimal effect both on IL-2 production and on spleen polyamine content. Irrespective of whether polyamine deprivation was started at an early or at an advanced stage of tumour growth, T-lymphocyte populations were restored to normal values, demonstrating that polyamine deprivation not only prevents tumour-induced immune suppression, but reverses established immunological disorders. In contrast to what was observed regarding IL-2 production by spleen cells and natural killer (NK) cell activity, the polyamine oxidase (PAO) inhibitor did not enhance the number of T lymphocytes. These findings are consistent with a direct effect of the polyamines on immune effector cell metabolism. They suggest an important role of the gastrointestinal polyamines and of PAO activity in the regulation of IL-2 production.

**Keywords:** polyamine; interleukin 2; T lymphocyte; immune suppression

The natural polyamines putrescine ($NH_2(CH_2)_4NH_2$), spermidine ($NH_2(CH_2)_3NH(CH_2)_4NH_2$) and spermine ($NH_2(CH_2)_3NH(CH_2)_4NH(CH_2)_3NH_2$) are ubiquitous cellular components that play an important role in proliferation and differentiation (Tabor and Tabor, 1984; Pegg, 1986; Heby, 1989). The first step in polyamine biosynthesis is the decarboxylation of ornithine by ornithine decarboxylase (ODC). Mitogen stimuli enhance ODC activity and polyamine levels; in fast-dividing cancer cells polyamine metabolism is increased (Pegg, 1988). The selective and irreversible inhibition of ODC by D,L-2-(difluoromethyl)ornithine (DFMO, Eflornithine) inhibits tumour cell proliferation in culture (Mamont et al, 1978). But in vivo, the anti-tumoral effect of DFMO was limited. This failure was partly because of the availability of polyamines from tissues, the diet and the intestinal microflora (Hessels et al, 1989; Sarhan et al, 1989). Tumour progression is prevented in animals treated with a polyamine-deficient diet, containing antibiotics for the decontamination of the gastro-intestinal tract, inhibitors of polyamine biosynthesis and the inter-conversion pathway (ODC and polyamine oxidase inhibitors) (Seiler et al, 1990; Hessels et al, 1991; Moulinoux et al, 1991*a,b*; Quemener et al, 1992).

The failure of the immune system to eliminate established cancer is partly because of the immune suppression induced by the tumour. In view of the importance of cell proliferation and differentiation in cell-mediated immune responses, polyamine deprivation, in addition to its inhibitory effect on tumour cell growth, could modulate immune responses of tumour-bearing mice. We have previously shown that the blockade of all major endogenous and exogenous polyamine sources, necessary to obtain an optimal tumour growth inhibition, normalizes the cytotoxic activity of natural killer (NK) cells (Chamaillard et al, 1993). Polyamines play important role in immune processes (see review by Seiler and Atanassov, 1994). As inhibition of polyamine biosynthesis by DFMO stimulates T-lymphocyte IL-2 production (Bowlin et al, 1987), it was of interest to know the influence of total polyamine deprivation on IL-2 production in vivo. As polyamine oxidation down-regulates IL-2 production in T lymphocytes (Flescher et al, 1989), it was important to evaluate the influence of a polyamine oxidase (PAO) inhibitor on the immune response in vivo. The aim of this study was to analyse the effects in vivo of the different components of polyamine deprivation on spontaneous IL-2 production and lymphocyte populations in mice grafted with the 3LL Lewis lung carcinoma.

*Received 12 September 1996*
*Revised 30 January 1997*
*Accepted 4 February 1997*

*Correspondence to:* V Catros-Quemener, GRETAC, URA CNRS 1529, Faculté de Médecine, 2 Avenue du Professeur L Bernard, Rennes 35043, France

### MATERIALS AND METHODS

### Chemicals and drugs

Metronidazole was from Rhône-Poulenc (Paris, France); penicillin and streptomycin from Biomérieux (Marcy-l'Etoile, France); [6-$^3$H]thymidine (sp. act.: 109 mCi mg$^{-1}$, 1 mCi ml$^{-1}$) from ICN Radiochemicals (Les Ulis, France); recombinant mouse IL-2 and monoclonal rat anti-mouse IL-4 antibody from Genzyme (Cambridge, USA); and RIA PGE2 kit from Amersham (Les Ulis, France). Standard rodent chow was from Pietrement (Provins, France). Polyamine-deficient chow (PDC), D,L-2(di-fluoromethyl)ornithine (DFMO; Eflornithine, MDL 71782) and

$N^1$,$N^4$-Bis-(2,3-butadienyl)-putrescine dihydrochloride (MDL 72527) were kind gifts from Marion Merrell Dow Research Institute (Strasbourg, France). Neomycin, anti-mouse CD4 R-phycoerythrin (anti-CD4-PE), anti-mouse CD8a quantum red (anti-CD8-QR), anti-mouse CD3 fluorescein isothiocyanate (anti-CD3-FITC), anti-mouse CD25 R-phycoerythrin (anti-CD25-PE) antibodies, mouse isotype controls and usual laboratory chemicals were from Sigma C (St Louis, MO, USA).

### Cell culture

Culture reagents were from Flow Laboratories (Irvine, CA, USA). The 3LL Lewis lung carcinoma cell line was maintained in RPMI-1640 medium supplemented with 2 mM L-glutamine, penicillin (100 U $ml^{-1}$), streptomycin (50 μg $ml^{-1}$) and 10% heat-inactivated fetal calf serum (supplemented medium) at 37°C under a 5% carbon dioxide atmosphere. The murine IL-2-dependent T-cell line (CTLL2) was maintained in the same medium, containing in addition 1 mM Hepes, 2.5 mM sodium pyruvate, 50 μM β-mercapto-ethanol and 0.8 U $ml^{-1}$ recombinant IL-2.

### Mice and tumours

Viable 3LL Lewis lung carcinoma cells ($5 \times 10^5$) were injected intramuscularly into the hind legs of 6- to 8-week-old F1-C57BL6/DBA2. Tumour volumes were measured as previously reported (Moulinoux et al, 1984). Animals were kept under standardized conditions: 20°C, 12 h light/dark cycle with food and water ad libitum. All animal procedures were performed strictly according to institutional guidelines.

### Treatments

Animals received ad libitum a polyamine-deficient chow (PDC) (Seiler et al, 1990), supplemented with neomycin (N) (2 g $kg^{-1}$) and metronidazole (M) (34 mg $kg^{-1}$) (diet designated NM-PDC) and with DFMO (30 g $kg^{-1}$) (drug containing polyamine-deficient chow: DC-PDC). When $N^1$,$N^4$-bis-(2,3-butadienyl)putrescine (MDL 72527, PAO inhibitor) (500 mg $kg^{-1}$) was added to DC-PDC, the diet was designated DC-PDC+. Controls received standard chow (SC) ad libitum; other groups received SC and DFMO or MDL 72527 in the drinking water at a dose corresponding to the DC-PDC+ treatment [3% (w/v) DFMO, 0.05% (w/v) MDL 72527]. All animals received drinking fluid ad libitum. For the composition and designation of various treatments see Table 1.

Treatments were given for 6 days either 1 week after tumour cell inoculation, when all mice had developed palpable tumours (0.1 $cm^3$ – early stage of tumour development), or 2 weeks after graft (0.8 $cm^3$ – advanced stage of tumour development) (Figure 1). Animals were sacrificed after 6 days of treatment. Healthy mice and untreated 3LL grafted mice were sacrificed at the same time in each experiment. All experiments were carried out three times.

### Preparation of spleen cells

Spleens were removed under sterile conditions, mechanically dissociated in RPMI-1640 medium, and filtered through sterile gauze to remove debris and to obtain single cell suspensions as previously described (Chamaillard et al, 1993). Erythrocytes were lysed by 0.17 M ammonium chloride, and the residual cells were washed and counted; viability was determined by trypan blue exclusion.

### Spontaneous IL-2 production

Spleen cells ($10^7$ cells $ml^{-1}$) were incubated in supplemented medium for 48 h at 37°C in 24-well tissue culture plates. Culture supernatants were collected and spontaneous IL-2 production was assessed by a bioassay measuring the proliferation of the CTLL2 cells according to the method of Gillis et al (1978). The CTLL2 cells were washed free of growth medium (exogenous IL-2) and resuspended in culture medium at a concentration of $4 \times 10^4$ cells $ml^{-1}$. Cells were distributed in 96-well microplates (0.1 ml) and 0.1 ml of different dilutions of supernatants were added. The cells were incubated for 48 h at 37°C – 5% carbon dioxide – and pulsed with 1 μCi $well^{-1}$ [6-$^3$H]thymidine for the final 8 h of culture. The labelled cells were harvested using a semiautomatic cell harvester (Skatron) and [6-$^3$H]thymidine incorporation measured using a beta-scintillation counter (Packard). IL-2 units were defined by the dilution of recombinant IL-2 required for 50% maximal proliferation. Because the proliferation of CTLL2 cell line is also IL-4 dependent, one assay was done in the presence of neutralizing anti-mouse IL-4 antibody (1.5 μg $ml^{-1}$) during the culture to distinguish IL-2 from IL-4 activity.

### Identification of splenic T-cell populations

The CD4 and CD8 T-cell populations were identified by double labelling, using anti-CD3-FITC/anti-CD4-PE and anti-CD3-FITC/anti-CD8-QR. The T-CD8$^+$ lymphocyte population, expressing the IL-2 (α-chain) receptor (CD25), was determined by triple labelling with anti-CD3-FITC/anti-CD8-QR/anti-CD25-PE.

Spleen cells ($0.5 \times 10^6$ cells) were suspended in PBS containing 1% bovine albumin and incubated for 30 min at 4°C before staining with 0.5 μg of the antibodies (or isotype control) for 30 min on ice. Cells were washed with 1 ml of phosphate-buffered saline (PBS) containing 0.5% bovine albumin, resuspended and fixed in 3% formaldehyde in PBS. Flow cytometric analysis was performed using a Coulter Epics Elite Flow Cytometer equipped with an argon laser (excitation wavelength at 488 nm, 15 mW). Green fluorescence was measured at 550 nm using long-pass dichroic and 525 nm bandpass filters, yellow fluorescence at 600 nm using long-pass dichroic and 575 nm bandpass filters and red fluorescence at 675 nm (bandpass filter). The mean of fluorescence was expressed in arbitrary units.

### Determination of prostaglandin $E_2$ production

A prostaglandin $E_2$ ($PGE_2$)-dependent immune suppressor phase has been described during the early phase of Lewis lung carcinoma growth (Ippoliti et al, 1985; Young et al, 1985). For this reason, the levels of $PGE_2$ were determined in plasma. An immunoassay using [$^{125}$I]$PGE_2$ as tracer was used. After dilutions (1:20), 500 μl of samples was acidified with 12.5 μl of 2 N hydrochloric acid (pH 3.5–4.5) and $PGE_2$ was extracted twice with 500 μl of ethyl acetate. Extracts were evaporated with a Speed Vac Concentrator (Savant). In this assay system, it is necessary to convert extracted $PGE_2$ into an oxime and $PGE_2$ was assessed by radioimmunoassay procedure using a standard calibration curve (0–1600 pg $ml^{-1}$).

© Cancer Research Campaign 1997

**Table 1** Composition and designation of various treatments

| Chow | Abbreviations | Drugs | | | |
|---|---|---|---|---|---|
| | | Neomycin | Metronidazole | DFMO | MDL 72527 |
| | | (g kg$^{-1}$ of drinking water or food) | | | |
| Standard chow | | | | | |
| | Controls | | | | |
| | DFMO | | | 30 | |
| | MDL 72527 | | | | 0.5 |
| Polyamine-deficient chow | | | | | |
| | NM-PDC | 2 | 0.034 | | |
| | DC-PDC | 2 | 0.034 | 30 | |
| | DC-PDC+ | 2 | 0.034 | 30 | 0.5 |

Animals fed with standard chow received drugs in drinking water. Animals fed with polyamine deficient chow received drugs in the food.

**Figure 1** Effect of polyamine deprivation on tumour growth and splenic T-cell populations. DC-PDC+ treatment was given either at an early stage of tumour development (1 week after tumour cell inoculation, when all mice had developed palpable tumours) or at an advanced stage of tumour development (2 weeks after graft). Animals were sacrificed after 6 days of treatment. Data are tumour volume and the percentage of cells expressing CD3/CD4 (T-CD4$^+$) and CD3/CD8 (T-CD8$^+$) differentiation markers (mean ± s.e.m. $n$ = 11–15). *Significantly different from untreated tumour-bearing mice 2 weeks after graft ($P$ < 0.05)

### Determination of polyamine concentrations

Spleen cells were prepared as described above after lysis of the erythrocytes. A total of $10^6$ spleen cells were sonicated in 1 ml of ice-cold 5% perchloric acid. After dansylation, perchloric extracts were submitted to HPLC separation as described previously (Moulinoux et al, 1984).

### Statistical analysis

The non-parametric Mann–Whitney ranking test was used to determine statistical significance when comparing treated 3LL mice with healthy or 3LL untreated mice.

### RESULTS

### Tumour growth inhibition

The growth of the 3LL carcinoma was significantly reduced by feeding mice for 6 days with a PDC containing antibiotics and inhibitors of polyamine metabolism (Table 2). Tumour growth inhibition due to polyamine deprivation was observed when treatment was started 1 or 2 weeks after graft (Table 2 and Figure 1).

### Effects of polyamine deprivation on weight and polyamine concentrations of the spleen and on $PGE_2$ plasma concentration

Two weeks after tumour cell inoculation, the spleen weight of mice with tumour was about twice that of controls. The polyamine concentration in the spleen cells of tumour-bearing mice was significantly increased (Table 2). After a treatment with DC-PDC or DC-PDC+, spleen weight and polyamine concentrations were restored to normal values. No significant difference was observed in the plasma $PGE_2$ levels of healthy or tumour-bearing mice (Table 2).

### Effects of polyamine deprivation on spontaneous IL-2 production by spleen cells

Lymphocyte stimulation with agents such as concanavalin A has a great effect on polyamine metabolism (for review see Seiler and Atanassov, 1994). Therefore, no mitogens were used in our study. Proliferation of the IL-2-dependent cell line CTLL2 was slow in the presence of the supernatant collected from the spleen-cell culture of tumour-bearing mice (Figure 2). By contrast, IL-2 production by the spleen cells of tumour-bearing mice treated with DC-PDC+ was similar to that of healthy mice (Figure 2). The presence of anti-mouse IL-4 antibody in our assay did not affect the proliferation of the murine CTLL2. As shown in Table 3, DC-PDC+ treatment increased IL-2 production in tumour-bearing mice 5.8 times. When drugs and/or components of DC-PDC+ were given separately, IL-2 levels increased but remained significantly lower than in healthy controls. Nevertheless, increase in IL-2 production by depletion of exogenous polyamines with NM-PDC, or by DC-PDC (without MDL 72527), was significant when compared with cancerous untreated mice (Table 3). DFMO did not add any effect to NM-PDC. When inhibitors of polyamine metabolism – DFMO or MDL 72527 – were given alone, the IL-2 production was slightly, but not significantly, increased compared with untreated tumour-bearing mice.

### Splenic T-lymphocyte populations

T-lymphocyte populations were decreased in the spleen of 3LL bearing mice compared with healthy mice (Figure 1). Two weeks after graft, CD3-positive cells (CD3$^+$) and T-CD4$^+$ lymphocytes were similarly reduced by 30% while the T-CD8$^+$ lymphocyte population exhibited a 50% reduction (Table 4). Consequently, the T-CD4$^+$/T-CD8$^+$ ratio in 3LL grafted mice increased compared with healthy controls (Table 4). A significant decrease in the T-CD8$^+$CD25$^+$ cell population was also observed (Table 5). However, the relative percentage of T-CD8$^+$CD25$^+$ cells in the total T-CD8$^+$ population remained unchanged: in treated or untreated cancerous mice around 22% of the T-CD8$^+$ cells were CD25 positive, 17% in healthy controls. No difference was observed in the intensity of the CD25 labelling (Table 5).

When DC-PDC or DC-PDC+ treatment was given at an early stage of tumour development (1 week after graft), T-CD4$^+$

© Cancer Research Campaign 1997

**Table 2** Effects of polyamine deprivation on tumour growth, on prostaglandin E2 ($PGE_2$) production, on spleen weight and splenic polyamine content.

| Treatment | Tumour volume ($cm^3$) | $PGE_2$ (pg $ml^{-1}$ plasma) | Spleen weight (mg) | Polyamine concentrations (pmol $10^{-6}$ spleen cells) | | |
|---|---|---|---|---|---|---|
| | | | | Putrescine | Spermidine | Spermine |
| Healthy controls | | 730 ± 100 | 75 ± 15 | 66 ± 22 | 241 ± 31 | 232 ± 33 |
| 3LL controls | 0.57 ± 0.38 | 850 ± 90 | 142 ± 44[a] | 103 ± 26[a] | 412 ± 91[a] | 328 ± 66[a] |
| DC-PDC | 0.25 ± 0.14[b] | 1190 ± 120 | 85 ± 13[b] | 69 ± 13[b] | 238 ± 23[b] | 265 ± 23 |
| DC-PDC+ | 0.22 ± 0.10[b] | 995 ± 155 | 85 ± 17[b] | 45 ± 11[b] | 232 ± 25[b] | 285 ± 29 |

Data were significantly different from healthy controls ([a]$P < 0.05$) and from untreated 3LL grafted mice ([b]$P < 0.05$). Treatments were started at an early stage of tumour development (1 week after graft). Data are the tumour volume, plasma concentration of $PGE_2$ and spleen weight after 6 days of treatment. The polyamine concentrations were determined on $10^6$ spleen cells. Mean of five values ± s.d.

**Figure 2** Proliferation of the IL-2-dependent CTLL2 cell line in the presence of spleen cell supernatants of polyamine deprived mice. Spleen cells were collected from healthy, treated (DC-PDC+) or untreated (3LL controls) tumour-bearing mice. Supernatants were diluted 4-, 8-, 16-, 32- or 64-fold. Data are the [$^3$H]-thymidine incorporation into CTLL2 cells, means ± s.d. ($n = 3$)

**Table 3** Influence of polyamine deprivation on splenic IL-2 production.

| Treatment | Interleukin 2 (mU $10^{-7}$ spleen cells) |
|---|---|
| Healthy controls | 124 ± 5 |
| 3LL controls | 17 ± 7[a] |
| MDL 72527 | 39 ± 9[a] |
| DFMO | 44 ± 9[a] |
| NM-PDC | 54 ± 10[a,b] |
| DC-PDC | 60 ± 11[a,b] |
| DC-PDC+ | 98 ± 12[b] |

Data were significantly different from healthy controls ([a]$P < 0.01$) and from untreated 3LL grafted mice ([b]$P < 0.05$). Mice were treated with MDL 72527, DFMO or with polyamine-deficient chow supplemented with neomycin and metronidazole (NM-PDC), and inhibitors of polyamine metabolism (DC-PDC, DC-PDC+) 1 week after graft. IL-2 production is expressed per $10^7$ spleen cells. Data are mean ± s.e.m. ($n = 9$).

lymphocyte populations were increased by 75% and T-CD8+ lymphocyte populations by 115% compared with untreated tumour-bearing mice (Table 4). Consequently, the T-CD4+/T-CD8+ ratio in mice treated with DC-PDC or DC-PDC+ decreased to reach normal value (Table 4).

Between the second and the third week after graft, the T-CD4+ lymphocyte population decreased by 20% (from 10.5% to 7.6%). In contrast, the T-CD8+ lymphocyte population remained unchanged (Figure 1). When polyamine deprivation was started 2 weeks after graft, at an advanced stage of tumour development (0.79 ± 0.08 $cm^3$), the tumour volume was not significantly increased (0.93 ± 0.01 $cm^3$) and T-CD4+ and T-CD8+ lymphocyte populations were completely restored to normal values after 6 days of treatment (Figure 1). Spleen cells from mice treated at an early or at an advanced stage of tumour development exhibited a significant increase in both T-CD4+ and T-CD8+ lymphocyte populations compared with untreated tumour-bearing mice (Figure 1).

### DISCUSSION

In the present study, we show that mice grafted with the 3LL carcinoma exhibit a dramatic immune suppression: spleen cells show both decreased spontaneous IL-2 production and decreased T-CD4+ and T-CD8+ lymphocyte populations. In addition, the polyamine content of the spleen was increased. By treating the animals with a polyamine-deficient diet containing antibiotics and inhibitors of polyamine metabolism (DC-PDC+), tumour growth was greatly reduced and the immune-abnormalities were completely reversed.

IL-2 production in spleen cells was enhanced in polyamine-depleted mice compared with untreated tumour-bearing mice. By reducing exogenous sources of the polyamines (polyamine-deficient diet containing antibiotics: NM-PDC), IL-2 was significantly overproduced. If selective specific inhibitors of polyamine metabolism (DFMO or MDL 72527) were given alone, IL-2 production was not significantly enhanced, and when DFMO was combined with NM-PDC (DC-PDC) no synergistic effect was observed. We have previously shown that the reduction in exogenous sources of polyamines (NM-PDC) exerts an anti-tumour effect and a significant improvement in NK cytotoxic activity without affecting polyamine concentrations in the tumour (Chamaillard et al, 1993). We confirm in this study that exogenous sources of polyamines can modulate the natural immune response.

The enhancement in IL-2 production was maximal when the PAO inhibitor (MDL 72527) was added to treatment (DC-PDC+), i.e. a total blockade of all major polyamine sources was necessary to obtain an optimal effect on spleen polyamine content and on IL-2 production. This was also evident for NK-cell activity (Chamaillard et al, 1993).

In contrast to what was observed with IL-2 and NK-cell activity, the effect of the PAO inhibitor on the enhancement of the T

 © Cancer Research Campaign 1997

**Table 4** T-lymphocyte populations in the spleen of tumour-bearing mice after polyamine deprivation (early stage of tumour growth)

| Treatment | CD3+ | T-CD4+ | T-CD8+ | T-CD4+/T-CD8+ |
|---|---|---|---|---|
| Healthy controls | 37.8 ± 1 | 14.6 ± 0.9 | 9.2 ± 0.5 | 1.7 ± 0.1 |
| 3LL controls | 27.5 ± 1.4[a] | 10.5 ± 0.7[a] | 4.6 ± 0.3[a] | 2.4 ± 0.3[a] |
| DC-PDC | 42.9 ± 2[b] | 18.3 ± 1.6[b] | 10.1 ± 0.7[b] | 2.0 ± 0.3 |
| DC-PDC+ | 43.5 ± 1.8[a,b] | 18.5 ± 1.7[b] | 10.4 ± 0.8[b] | 1.8 ± 0.2[b] |

Significantly different from healthy mice ([a]$P < 0.005$) and from 3LL grafted mice ([b]$P < 0.005$). Treatments started at an early stage of tumour development (1 week after graft) and lymphocytes were analysed after 6 days of polyamine deprivation. The CD4 and CD8 T cells were identified by double labelling: CD3/CD4 (T-CD4+) and CD3/CD8 (T-CD8+). Data are the percentage of cells expressing differentiation markers (mean ± s.e.m. $n = 12$).

**Table 5** Expression of the IL-2 receptor of the T-CD8+ lymphocytes after polyamine deprivation.

| Treatment | T-CD8+CD25+ | T-CD8+CD25+/T-CD8+ | Mean fluorescence |
|---|---|---|---|
| Healthy controls | 1.6 ± 0.25 | 17.4 ± 2.6 | 1.9 ± 0.1 |
| 3LL controls | 0.8 ± 0.1[a] | 21.2 ± 3.6 | 2.1 ± 0.1 |
| DC-PDC | 2.1 ± 0.3[b] | 22.4 ± 2.9 | 2.1 ± 0.1 |
| DC-PDC+ | 2.0 ± 0.2[b] | 22.3 ± 3.5 | 2.2 ± 0.2 |

Significantly different from healthy controls with [a]$P < 0.05$ and from untreated 3LL grafted mice with [b]$P < 0.05$. Treatments started at an early stage of tumour development (1 week after graft) and lymphocytes were analysed after 6 days of polyamine deprivation. Data are the percentage of T-CD8+ cells expressing the IL-2 receptor (determined by triple labelling T-CD8+CD25+), and the mean of fluorescence of the CD25-labelling (arbitrary units). Means ± s.e.m. ($n = 12$)

lymphocytes was not important: the augmentation of T-CD4+ and T-CD8+ cells was similar in DC-PDC– or DC-PDC+-treated mice.

It is noteworthy that in tumour-bearing mice the decrease in T-CD4+ and T-CD8+ lymphocyte populations is time and population dependent. The most marked decrease (50% reduction) was observed 3 weeks after graft for T-CD4+ and 2 weeks after graft for the T-CD8+ population. This decrease is not due to lack of IL-2 receptor expression as in tumour bearing mice 22% of T-CD8+ cells were CD25 positive compared with 17% in healthy controls.

Irrespective of whether or not polyamine deprivation started at an early or at an advanced stage of tumour growth (i.e. at a time when immune suppression was established), T-CD4+ and T-CD8+ lymphocyte populations were completely restored to normal values. This observation demonstrates that polyamine deprivation not only prevents tumour-induced immune suppression, but also is capable of reversing established immunological disorders. However, the percentage of T-CD8+ cells expressing the IL-2 receptor in mice treated with DC-PDC or DC-PDC+ remained unchanged. Thus, our treatment did not induce any specific activation of cytotoxic T lymphocytes, but it enhanced their number.

One may speculate that the decrease in IL-2 production in tumour-bearing mice is due to a relative decrease in the T-CD4+ lymphocyte population owing to the invasion by other cells into the spleen (splenomegaly). This assumption is not correct, as the IL-2 production per $10^6$ T-CD4+ cells was 85 mU for healthy controls but only 16 mU for 3LL tumour-grafted mice. In mice deprived of polyamines we found 50 mU IL-2 per $10^6$ T-CD4+ cells.

The immune suppression observed in tumour-bearing animals could be indirectly mediated by the induction of suppressor cells, or directly by the secretion of immune-suppressive factors by tumour cells. The induction of immune-suppressor cells by tumour-derived colony-stimulating factors has been reported (Young and Wright, 1992; Oghiso et al, 1993). During the early phase of Lewis lung carcinoma growth, tumour cells stimulate macrophages suppressing T-cell competence. This immune-suppressor phase is $PGE_2$ dependent (Ippoliti et al, 1985; Young et al, 1985). In our study, changes in $PGE_2$ plasma levels were not observed during the early stage of tumour growth. Concerning the immunosuppressive factors secreted by tumour cells, polyamine deprivation treatment could have direct or indirect effects. As polyamines play an important role in the proliferation and differentiation processes, and as inhibition of polyamine metabolism decreased protein synthetic activity (Rudkin et al, 1984; Hölttä, 1985), polyamine deprivation could, by reducing tumour growth, reduce the levels of immune-suppressive factors. However, the reduction in the tumour burden had no major contribution to immune restoration, as the T-CD8+ populations were significantly different in treated and untreated mice with a similar tumour volume (0.8–0.9 $cm^3$, see Figure 1). Our findings show that polyamine deprivation not only prevents the decrease in T-lymphocyte populations but also reverses the tumour-induced immune suppression; they are not in favour of an indirect effect proportional to the tumour burden. One cannot exclude an influence of polyamine deprivation on tumour cell metabolism.

In our opinion the qualitative and quantitative modulation of T-lymphocyte populations could be due to a direct effect of polyamine deprivation on immune effector cells. Immune suppression has been reported to be associated with increased polyamine levels (Flescher et al, 1992; Thomas et al, 1992). Thomas et al observed in MRL-lpr/lpr lupus-prone mice, aberrations in thymic maturation of T cells and increased putrescine and spermidine levels. In this model the use of DFMO abolished the increase in T-cell polyamine levels, and increased the number of cells with the correct surface markers (Thomas et al, 1992).

Inhibition of T-lymphocyte IL-2 production in mice grafted with the 3LL Lewis lung carcinoma could be a consequence of the high

© Cancer Research Campaign 1997

polyamine levels in the spleen. Thomas et al (1993) reported the blocking of the transmembrane $Ca^{2+}$ influx in CD4-positive cells by putrescine, a process essential for IL-2 production (Mills et al, 1985). This observation supports the idea that polyamine deprivation could be directly responsible for the enhancement of IL-2 production by helper cells in the spleen. DC-PDC+ was the most effective treatment, in agreement with the most complete depletion of putrescine. The PAO inhibitor participated in the enhancement of IL-2 production. In agreement with this observation are data showing that the products of PAO activity induce a decrease in both IL-2 mRNA levels and IL-2 production via suppression of mitogen-induced transmembrane signalling (Flescher et al, 1994).

It seems very likely that polyamine deprivation in vivo has a direct effect on the immune effector cells metabolism, as was shown to be the case in vitro. As the reduction of exogenous sources of polyamines exerts an anti-tumour effect without affecting tumour polyamine content, it appears likely that the enhancement of spontaneous IL-2 production, NK-cell activity and T-lymphocyte populations contributes to the anti-tumoral effect of the polyamine deprivation regimen. As numerous immune cells are sensitive to IL-2, polyamine deprivation could be of considerable interest in combination with anti-tumoral immunotherapy trials.

### ACKNOWLEDGEMENTS

We thank Professor N Seiler for discussion of the data. This work was supported by grants from the Centre National de la Recherche Scientifique and Institut National de la Santé et de la Recherche Médicale (INSERM CRE 930505). L Chamaillard was a recipient of a grant from the Association pour la Recherche sur le Cancer (ARC).

### REFERENCES

Bowlin TL, McKown BJ and Sunkara PS (1987) The effect of α-difluoromethylornithine, an inhibitor of polyamine biosynthesis, on mitogen-induced interleukin 2 production. *Immunopharmacology* **13**: 143–147

Chamaillard L, Quemener V, Havouis R and Moulinoux J-PH (1993) Polyamines deprivation stimulates natural killer cell activity in cancerous mice. *Anticancer Res* **13**: 1027–1034

Flescher E, Bowlin TL and Talal N (1989) Polyamine oxidation down-regulates IL-2 production by human peripheral blood mononuclear cells. *J Immunol* **142**: 907–912

Flescher E, Bowlin TL and Talal N (1992) Regulation of IL-2 production by mononuclear cells from rheumatoid arthritis synovial fluids. *Clin Exp Immunol* **87**: 435–437

Flescher E, Ledbetter JA, Schieven GL, Vela-Roch N, Fossum D, Dang H, Ogawa N and Talal N (1994) Longitudinal exposure of human T lymphocytes to weak oxidative stress suppresses transmembrane and nuclear signal transduction. *J Immunol* **153**: 4880–4889

Gillis S, Ferm MM, Ou W and Smith KA (1978) T cell growth factor: parameters of production and a quantitative microassay for activity. *J Immunol* **120**: 2027–2032

Heby O (1989) Polyamines and cell differentiation. In *The Physiology of Polyamines*, Bachrach U and Heimer YM (eds) pp. 83–94. CRC Press: Boca Raton (FL)

Hessels J, Kingma AW, Ferwerda H, Keij J, Berg GAVD and Muskiet FAJ (1989) Microbial flora in the gastrointestinal tract abolishes cytostatic effects of α-difluoromethylornithine in vivo. *Int J Cancer* **43**: 1155–1164

Hessels J, Kingma AW, Muskiet FAJ, Sarhan S and Seiler N (1991) Growth inhibition of two solid tumors in mice, caused by polyamine depletion, is not attended by alterations in cell-cycle phase distribution. *Int J Cancer* **48**: 697–703

Hölttä E (1985) Polyamine requirement for polyribosome formation and protein synthesis in human lymphocytes. In *Recent Progress in Polyamine Research*, Selmeci L, Brosnan ME and Seiler N (eds), pp. 137–150. Akadémiai Kiadò: Budapest

Ippoliti F, Sezzi ML, Bellelli L, Naso G and Pontieri GM (1985) Immunosubversive role of $PGE_2$ in tumor bearing mice. *Boll Ist sieroter milan* **64**: 25–34

Mamont PS, Duchesne M-C, Grove J and Bey P (1978) Anti-proliferative properties of DL-α-difluoromethyl ornithine in cultured cells. A consequence of the irreversible inhibition of ornithine decarboxylase. *Biochem Biophys Res Commun* **81**: 58–66

Mills GB, Cheung RK, Grinstein S and Gelfand EW (1985) Increase in cytosolic free calcium concentration is an intracellular messenger for the production of interleukin 2 but not for expression of the interleukin 2 receptor. *J Immunol* **134**: 1640–1643

Moulinoux J-PH, Quemener V, Larzul J-J, Calve ML, Roch A-M, Toujas L and Quash G (1984) Red blood cell polyamines in mice bearing the Lewis lung carcinoma (3LL) and in patients with bronchopulmonary cancers. *Int J Cancer* **34**: 277–281

Moulinoux J-PH, Darcel F, Quemener V, Havouis R and Seiler N (1991*a*) Inhibition of the growth of U-251 human glioblastoma in nude mice by polyamine deprivation. *Anticancer Res* **11**: 175–180

Moulinoux J-PH, Quemener V, Cipolla B, Guillé F, Havouis R, Martin C, Lobel B and Seiler N (1991*b*) The growth of MAT-LyLu rat prostatic adenocarcinoma can be prevented in vivo by polyamine deprivation. *J Urol* **146**: 1408–1412

Oghiso Y, Yamada Y, Ando K, Ishihara H and Shibata Y (1993) Differential induction of prostaglandin $E_2$-dependent and independent immune suppressor cells by tumor-derived GM-CST and M-CSF. *J Leukoc Biol* **53**: 86–92

Pegg AE (1986) Recent advances in the biochemistry of polyamines in eukaryotes. *Biochem J* **234**: 249–262

Pegg AE (1988) Polyamine metabolism and its importance in neoplastic growth and as a target for chemotherapy. *Cancer Res* **48**: 759–774

Quemener V, Moulinoux J-PH, Bergeron C, Darcel F, Cipolla B, Denais A, Havouis R, Martin C and Seiler N (1992) Tumor growth inhibition by polyamine deprivation. In *Polyamines in the Gastrointestinal Tract*, Dowling, Folsch and Loser (eds), pp. 375–385. Kluwer Academic Press: Lancaster, UK

Rudkin BB, Mamont PS and Seiler N (1984) Decreased protein-synthetic activity is an early consequence of spermidine depletion in rat hepatoma tissue-culture cells. *Biochem J* **217**: 731–741

Sarhan S, Knödgen B and Seiler N (1989) The gastrointestinal tract as polyamine source for tumor growth. *Anticancer Res* **9**: 215–224

Seiler N and Atanassov CL (1994) The natural polyamines and the immune system. In *Progress in Drug Research*, Jucker E (ed.), pp. 87–141. Birkhäuser: Basle Switzerland

Seiler N, Sarhan S, Grauffel C, Jones R, Knödgen B and Moulinoux J-PH (1990) Endogenous and exogenous polyamines in support of tumor growth. *Cancer Res* **50**: 5077–5083

Tabor CW and Tabor H (1984) Polyamines. *Ann Rev Biochem* **53**: 749–790

Thomas TJ, Gunnia UB and Thomas T (1992) Reversal of the abnormal development of T cell subpopulations in the thymus of autoimmune MRL-*lpr/lpr* mice by a polyamine biosynthesis inhibitor. *Autoimmunity* **13**: 275–283

Thomas T, Gunnia UB, Yurkow EJ, Seibold JR and Thomas TJ (1993) Inhibition of calcium signalling in murine splenocytes by polyamines: differential effects on CD4 and CD8 T-cells. *Biochem J* **291**: 375–381

Young MRI and Wright MA (1992) Myelopoiesis-associated immune suppressor cells in mice bearing metastatic Lewis lung carcinoma tumors: γ interferon plus tumor necrosis factor α synergistically reduces immune suppressor and tumor growth-promoting activities of bone marrow cells and diminishes tumor recurrence and metastasis. *Cancer Res* **52**: 6335–6340

Young MR, Newby M and Meunier J (1985) Relationships between morphology, dissemination, migration, and prostaglandin $E_2$ secretion by cloned variants of Lewis lung carcinoma. *Cancer Res* **45**: 3918–3923

© Cancer Research Campaign 1997